# Record-low Antarctic sea ice in 2023 increased ocean heat loss and storms

**DOI:** 10.1038/s41586-024-08368-y

**Published:** 2024-12-18

**Authors:** Simon A. Josey, Andrew J. S. Meijers, Adam T. Blaker, Jeremy P. Grist, Jenny Mecking, Holly C. Ayres

**Affiliations:** 1 National Oceanography Centre, Southampton, UK; 2 British Antarctic Survey, Cambridge, UK; 3 University of Reading, Reading, UK

**Keywords:** Physical oceanography, Physical oceanography

## Abstract

Recent Antarctic sea-ice decline is a substantial source of concern, notably the record low in 2023 (ref. ^1^). Progress has been made towards establishing the causes of ice loss^1–5^ but uncertainty remains about its consequences for ocean–atmosphere interaction. Resolution of this uncertainty is important as ice decline can substantially alter surface heat loss and thus the ocean and atmosphere^6^. Here we show that the strongest winter 2023 ice-retraction regions provide an important new source of turbulent ocean heat loss to the atmosphere in wintertime. Ice concentration in these regions (located primarily in the Weddell, Bellingshausen and Ross seas) is reduced by up to 80% and is accompanied by an unprecedented doubling of mid-winter ocean heat loss. Also, there is a phase shift in the time of peak heat loss from late April to mid-June, with weaker than normal heat loss in austral autumn. The winter surface-heat-loss intensification is accompanied by substantial changes on both sides of the ocean–atmosphere interface. These include increases in atmospheric-storm frequency and surface-heat-loss-driven dense water formation, although the implications of the densification for broader processes such as Antarctic bottom water formation remain unclear. Our results reveal that the 2023 Antarctic sea-ice loss has substantially modified air–sea interaction in the Southern Ocean and motivate in-depth analysis of the wider climate-system impacts.

## Abstract (web summary)

The record-low Antarctic sea-ice decline in 2023 substantially altered Southern Ocean–atmosphere interaction leading to unprecedented wintertime turbulent ocean heat loss to the atmosphere, enhanced storminess and increased dense water formation.

## Main

Antarctic sea-ice extent has experienced notable reductions starting in 2016, before which it exhibited a weak, regionally variable multidecadal increase^7,8^. In 2022, ice extent was again substantially lower than normal^9^. However, these years were eclipsed by 2023, which set a new low for February ice extent and experienced continued exceptionally low ice cover in subsequent months. This led to a June sea-ice-extent anomaly of 2.33 million square kilometres, twice as large as the previous June record^1^. The severity of sea-ice decline is a substantial concern as it has a wide range of physical and biological impacts that include ocean warming leading to enhanced ice-shelf melt^10,11^, weakening of the Southern Ocean carbon sink^12^ and population reduction leading to potential colony extinction in penguins^13^. Furthermore, climate model analyses have shed light on the wider multidecadal consequences of projected ice loss, which extend to the tropics and the Northern Hemisphere^6,14^.

The focus so far has been on ice-loss drivers, which include melt from below owing to increasing ocean temperatures^1,5^ and above from a warmer atmosphere^2–4,15,16^, with further contributions from wind-driven sea-ice advection^17^. The potential for fundamental changes in ocean heat loss to the atmosphere following the extensive removal of the winter insulating sea-ice cap have yet to be determined. Here we analyse winter 2023 and identify a pattern of extreme ocean heat loss with main centres in the outer Weddell and Ross seas, Bellingshausen Sea and the ocean north of Enderby Land; the heat loss in each area is unprecedented in the modern period (1990–2023). Our goal is to understand how air–sea heat exchange is changing in the absence of ice and explore the wider implications for the ocean and atmosphere.

Our focus is the most severe ice-decline period (June–July) in winter 2023 (referred to hereafter as JJ23). We contrast JJ23 heat loss with (1) June–July 1990–2015 (hereafter JJ9015), which represents conditions before the first sharp ice decline in 2016, and (2) June–July 2016–2022 (hereafter JJ1622), which spans several previous but less extreme low ice cover years. Then, we put the winter changes in the context of the seasonal cycle and identify a phase shift in the timing of peak heat loss, which previously occurred in late April but shifted to mid-June in 2023. Finally, we identify consequences of the increased winter heat loss, which include substantial changes to ocean water-mass formation and atmospheric-storm frequency.

## Ice reduction impacts on ocean heat loss

**Fig. 1:**
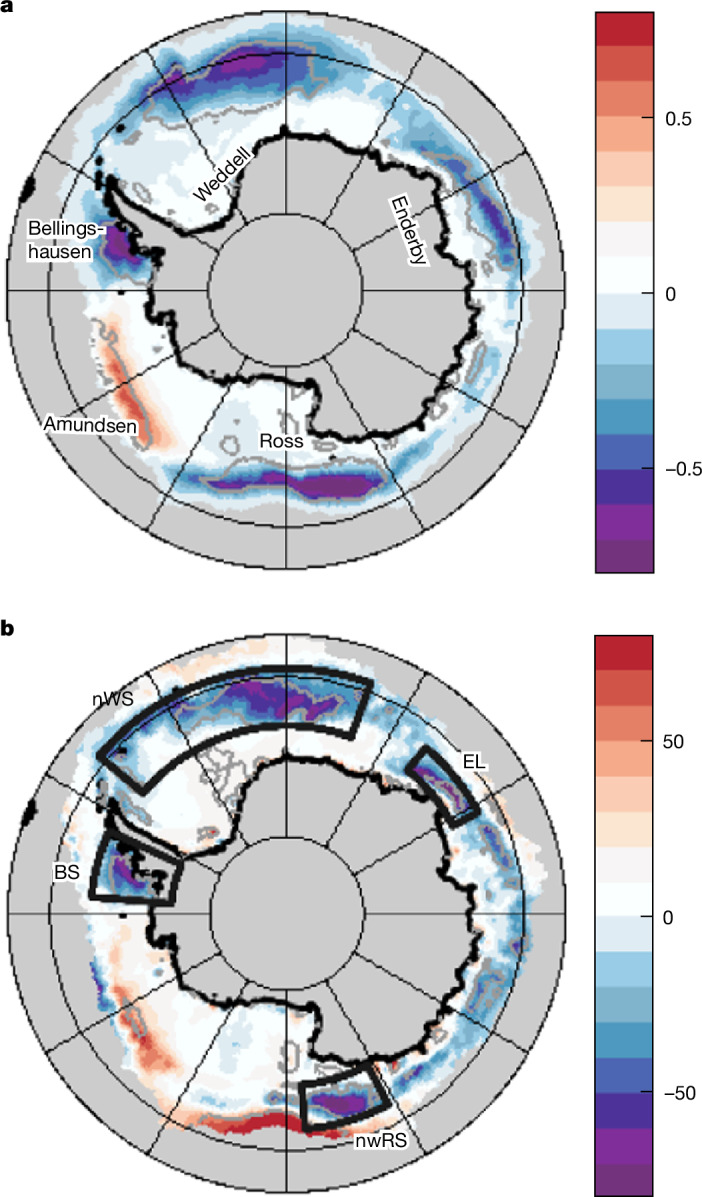
Sea-ice and surface net heat-flux anomalies reveal increased ocean heat loss to the atmosphere in regions of JJ23 ice decline. **a**, Sea-ice concentration anomaly (difference of fractional sea-ice concentration in JJ23 from the 1991–2020 climatological June–July mean). **b**, Net heat-flux anomaly (W m^−2^) within the sea-ice zone (climatological June–July sea-ice concentration > 0%). Boxes show the four regions used for subsequent analysis: BS, Bellingshausen Sea; EL, Enderby Land; nwRS, northwestern Ross Sea; nWS, northern Weddell Sea. Grey contours show 95% significance level of 2023 anomalies compared with the climatological baseline. Anomalies are determined with respect to 1991–2020 and fields are from the ERA5 reanalysis^27^ (see Methods). For context, the mean (rather than anomalous) climatological and JJ23 heat-flux fields are shown in Extended Data Fig. 3.

**Extended Data Fig. 1:**
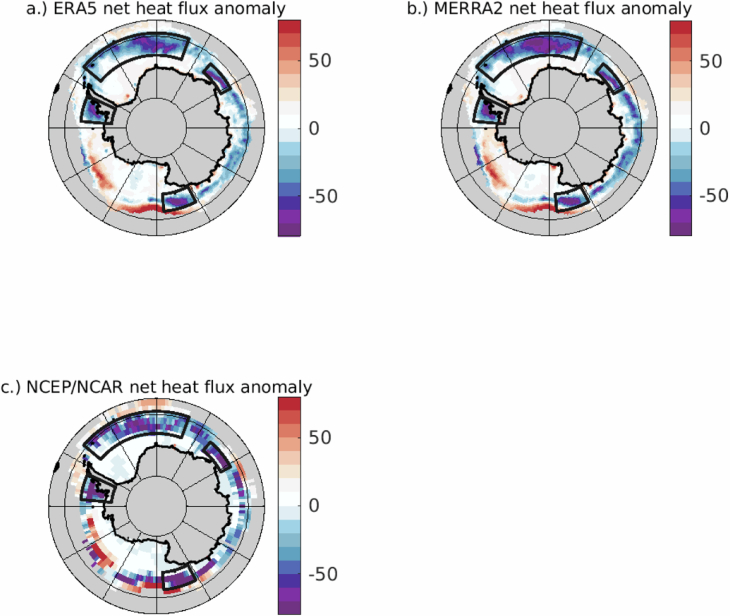
Intercomparison of surface net heat-flux anomalies shows robust pattern in JJ23 for the ERA5, MERRA-2 and NCEP/NCAR reanalyses. Net heat-flux anomaly (difference of JJ23 mean from the 1991–2020 climatological June–July mean) for ERA5 (**a**), MERRA-2 (**b**) and NCEP/NCAR (**c**) (W m^−2^). Boxes show the four regions used for the main-text analysis.

Three notable hotspots of anomalously low sea-ice cover are evident in JJ23 (Fig. 1a; strongest reductions up to 80%) located in the outer Weddell and Ross seas and the entire Bellingshausen Sea. Furthermore, sea-ice cover is 50% below normal in the ocean north of Enderby Land. The Amundsen Sea is the only region with a substantial increase (50% above normal). A substantial change in ocean–atmosphere interaction is observed in all four ice-decline regions with anomalous net heat loss exceeding −70 W m^−^^2^ (Fig. 1b; a very similar pattern of change is obtained with a second reanalysis, MERRA-2; Extended Data Fig. 1). At an example location (64° S, 0° E) in the outer Weddell Sea, heat loss has more than doubled from −57 W m^−^^2^ (JJ9015) to −132 W m^−^^2^ (JJ23). The stronger heat loss is consistent with ocean surface exposure in previously ice-covered regions, as this enables intense turbulent (sensible plus latent) heat flux to the atmosphere. There is a close relationship between the enhanced heat loss and reduced ice cover, but the match is not as exact as other factors, including both wind speed and sea–air temperature/humidity difference, also play a role.

**Fig. 2:**
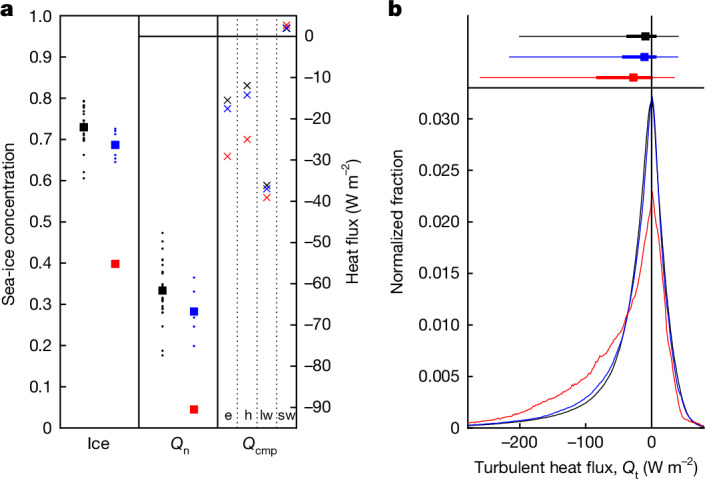
Comparison of sea-ice and surface heat flux between the pre-ice-decline and ice-decline periods shows a step change in conditions in winter 2023. **a**, Sea-ice concentration (Ice), net heat flux (*Q*_n_) and heat-flux components (*Q*_cmp_) averaged over the ice-decline regions (area-weighted mean taken over the four boxes in Fig. 1b). For Ice and *Q*_n_, black squares (points) show JJ9015 means (June–July means for individual years in 1990–2015). Likewise for JJ1622 (blue) and JJ23 (red). For *Q*_cmp_, crosses indicate JJ9015 (black), JJ1622 (blue) and JJ23 (red) means. Individual year points are omitted for ease of comparison. Components are labelled as follows: e, latent; h, sensible, lw, longwave, sw, shortwave. **b**, Bottom, normalized distribution of daily turbulent (latent + sensible) heat loss for the four ice-decline regions combined (distributions are formed from individual ERA5 grid-cell values): JJ9015 (black), JJ1622 (blue) and JJ23 (red). Top, median values (squares), 25–75% range (thick bar) and 5–95% range (thin bar).

**Extended Data Fig. 2:**
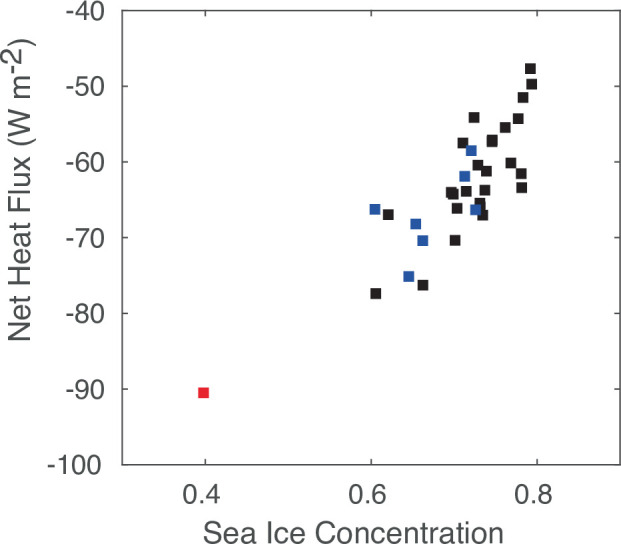
Net air–sea heat flux shows increasing ocean heat loss as sea-ice concentration decreases. Values shown are ERA5 area-weighted means taken over the four boxed ice-decline regions defined in the main text. Colours denote June–July means for 1990–2015 (black), 2016–2022 (blue) and 2023 (red).

The June–July ice area summed over the four main boxed regions of decline (Fig. 1b) is 1.82 × 10^12^ km^2^ in 2023, a reduction by nearly a half compared with 1990–2015, 3.35 ± 0.22 × 10^12^ km^2^. Consistent with the decline, the four-box mean net ocean heat loss is −91 W m^−^^2^ in JJ23, substantially stronger than both JJ9015 (−62 ± 7 W m^−^^2^) and JJ1622 (−67 ± 5 W m^−^^2^) (Fig. 2). JJ1622 includes previous ice-decline years but the main ice reductions in these years occurred in austral summer^3,9,17^ and not winter; hence, the JJ1622 heat loss is only marginally stronger than that in JJ9015. A clear relationship showing increasing heat loss with ice decline is evident in Extended Data Fig. 2 and this figure further emphasizes the exceptional nature of ocean–atmosphere interaction in JJ23. Decomposition of the net heat flux reveals that the stronger JJ23 heat loss is primarily because of increasingly negative sensible and latent heat fluxes (Fig. 2a, right panel). The four-box mean turbulent heat flux has doubled from −27 ± 7 W m^−^^2^ in JJ9015 to −54 W m^−^^2^ in JJ23. The changes in the radiative flux terms (longwave, shortwave) are small by comparison.

Intense heat loss can occur at smaller spatial and temporal scales than the box averages considered previously. The distribution of daily mean turbulent heat flux at the grid-cell level (order 10 × 25 km; Fig. 2b) reveals an extended intense heat-loss tail in JJ23 compared with JJ9015 and JJ1622. The proportion of heat-loss events <−100 W m^−^^2^ doubles from 9.0% (JJ9015)/10.8% (JJ1622) to 20.7% (JJ23). In terms of distribution measures, the lower limit of the interquartile range has strengthened by nearly 50 W m^−^^2^ from −38 W m^−^^2^ in JJ9015 to −84 W m^−^^2^ in JJ23. Strong heat-loss events are key to initiating ocean convection^18^ and influence atmospheric storms^19^. Thus, the shift towards more frequent extreme events in 2023 has the potential to substantially affect both the ocean and the atmosphere.

## Phase shift of maximum ocean heat loss

**Fig. 3:**
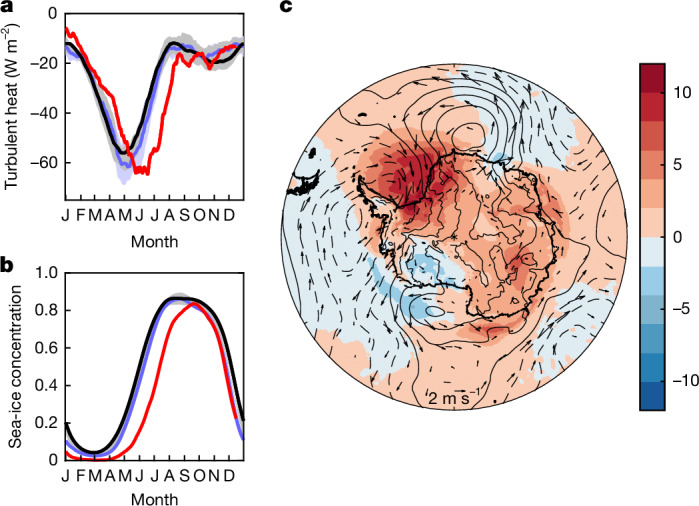
Substantial shifts in the timing of 2023 peak ocean heat loss and sea-ice concentration, and atypical pre-winter warm-air incursions. **a**, Daily mean turbulent heat flux averaged over the four boxes in Fig. 1b: 1990–2015 (black), 2016–2022 (blue) and 2023 (red). All values smoothed with a 31-day running mean. Shading: ±1 standard deviation of individual daily values for 1990–2015 (grey) and 2016–2022 (blue). **b**, As **a** but for sea-ice concentration. **c**, Anomaly fields for April 2023 sea-level pressure (contours, zero and positive values solid, negative values dashed, interval 2 mb), 2 m air temperature (coloured field, °C) and wind speed (vectors, reference horizontal wind vector shows a 2 m s^−1^ anomaly). All data are from the ERA5 reanalysis. Anomalies taken relative to April 1991–2020.

Time series of the turbulent air–sea heat flux averaged over the ice-decline regions reveal a substantial phase shift of 2023 ocean heat loss relative to earlier years (Fig. 3a). Peak turbulent heat loss in 2023 occurs on 18 June compared with 30 April (1990–2015) and 8 May (2016–2022), that is, the timing of maximum heat supply to the atmosphere is delayed by more than a month. It is also noticeable that heat loss throughout April 2023 is weaker than the earlier years. This favours the slow rate of ice regrowth that is evident in April 2023 (Fig. 3b) and is a contributing factor to the low ice concentration in JJ23. The large-scale context for this slow regrowth is shown in Fig. 3c, which reveals intrusion of warm air into the main ice-decline regions. The warmer-than-normal near-surface air temperature is particularly noticeable in the Weddell Sea. It has the potential to limit sea-ice growth by reducing the sea–air temperature gradient and thus contribute to low ice conditions in April that can persist and enable the strong heat loss subsequently seen in June–July. Also, the strong northerly wind component is likely to inhibit sea-ice expansion and further contribute to the lower winter ice area in 2023. The ice concentration recovers in August but still remains below the 1990–2015 mean for the rest of 2023, potentially priming the 2023 decline regions for further low ice concentrations in 2024. Considering the whole year, the 2023 annual mean turbulent heat flux over the ice-decline regions is −29 W m^−^^2^, which is similar to the corresponding values of −26 ± 2 W m^−^^2^ for JJ9015 and −27 ± 2 W m^−^^2^ for JJ1622, that is, the phase shift tends to compensate in the annual mean and the main signals of interest are those in April and June–July.

## Consequences for ocean and atmosphere

**Fig. 4:**
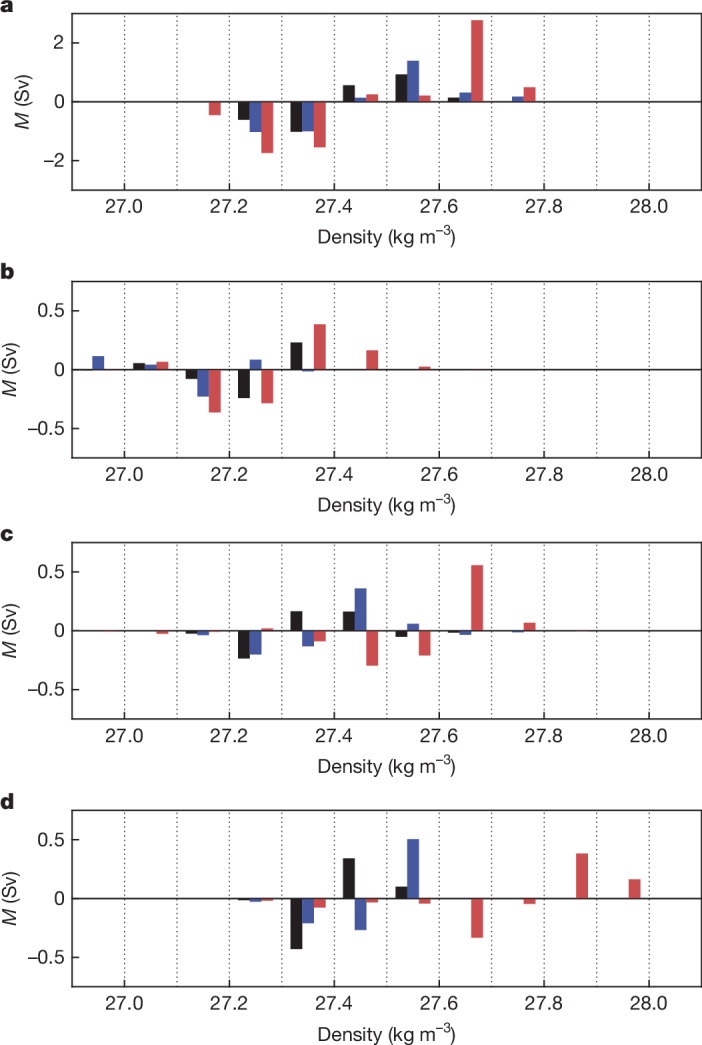
Surface flux contribution to water-mass formation (*M*) in the pre-ice-decline and ice-decline periods reveals increased formation at high densities in JJ23. Values are shown in 0.1 kg m^−^^3^ class intervals for JJ9015 (black), JJ1622 (blue) and JJ23 (red). Units are Sv, in which 1 Sv = 10^6^ m^3^ s^−^^1^. **a**, Northern Weddell Sea. **b**, Bellingshausen Sea. **c**, Northwestern Ross Sea. **d**, Enderby. Note the difference in *y*-axis scales between **a** and **b**–**d**. Values are derived from the ERA5 reanalysis. See also Extended Data Fig. 4 for MERRA-2-derived values, which show similar results.

The marked increase in JJ23 surface heat loss may affect both the ocean and the atmosphere. Here we focus on potential consequences for ocean water-mass formation and atmospheric-storm activity. Using water-mass-transformation theory (see Methods), we have determined the surface flux contribution to the amount of water formed in different density intervals (Fig. 4, see also Extended Data Fig. 4). As a result of the increased heat loss and new exposure to the atmosphere of denser water masses, a stronger high-density contribution to June–July water-mass formation is observed in 2023 than both 1990–2015 and 2016–2022. Focusing on the northern Weddell Sea, in JJ23, the contribution peaks at a higher density range (27.6–27.7 kg m^−^^3^) and exceeds the corresponding values for JJ9015 and JJ1622 by more than a factor of 10. The Bellingshausen, northwestern Ross and Enderby boxes also show substantial increases at high densities, with formation occurring for the first time in density classes up to 28.0 kg m^−^^3^. It should be noted that Fig. 4 does not include potential extra effects from brine rejection associated with ice formation as further data on ice thickness, not reliably available from observations, would be required to calculate the ice contribution.

A full determination of the consequences of reduced ice cover for water-mass formation requires an ocean-state-estimate analysis that can include all relevant terms (heat, net evaporation, runoff, ice melt, brine rejection, mixing). Such a study was carried out for the wider Southern Ocean for the pre-ice-decline period 2005–2010 (ref. ^20^). Ocean-state estimates^20,21^ do not yet cover 2023 but, when available, are likely to yield valuable insights into recent changes in water-mass formation within the ice-decline regions, as well as the wider Southern Ocean air–sea interaction regime^22^. The increased surface heat-loss contribution to high-density water-mass formation in JJ23 that we have identified will potentially result in a denser class of Antarctic surface water^23^ (ASW). Although the location of the modified ASW formation is far from the densest water-formation sites at the Antarctic shelf, mixing between this new ASW vintage and underlying circumpolar deep water (CDW) may substantially modify the properties of the CDW. In the Weddell Sea, the cyclonic circulation of the gyre is known to bring modified CDW onto the shelf and thus has the potential through advection and further mixing of the modified CDW to influence the properties and rate of Antarctic bottom water (AABW) formation^24^. Ocean-state-estimate analysis is urgently needed to establish the extent and timescale on which this mechanism operates and thus whether the massive decline of offshore winter sea ice in 2023 will ultimately affect AABW production and export from the Southern Ocean.

In subsequent austral winters that experience ice loss at 2023 levels, we anticipate repeated episodes of new dense ASW formation and export of modified CDW to the shelf. AABW is central to the Southern Ocean uptake of anthropogenic heat and carbon^24^ and influences the wider global ocean circulation. Thus, advection onto the shelves of modified water masses formed in the newly ice-free outer Weddell Sea may have far-reaching consequences. Similarly, the properties of CDW upwelled onto the coast in the Amundsen and Bellingshausen seas are critical for driving ice-shelf melt^10,11^, so changes in air–sea fluxes in these sectors may have further serious impacts.

**Fig. 5:**
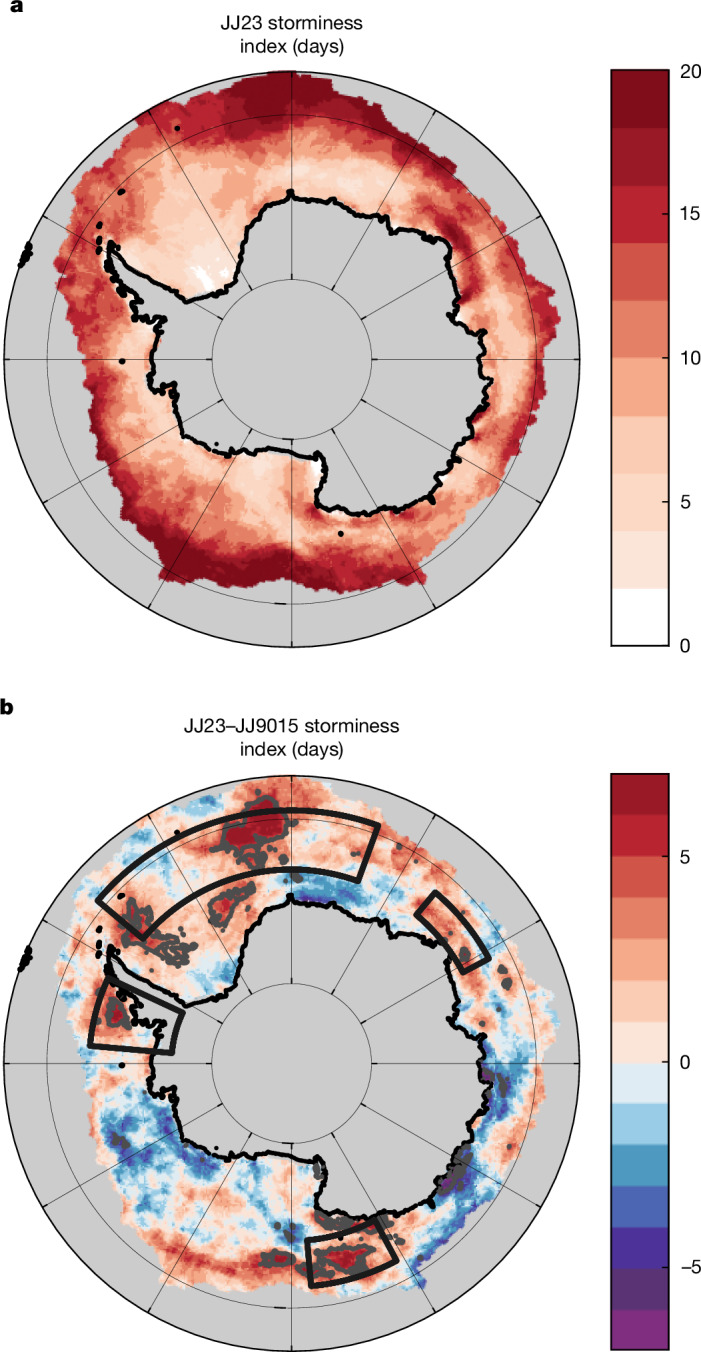
Frequency of storms within the Antarctic sea-ice region shows the increase in winter 2023 storminess relative to 1990–2015. **a**, Storminess index (number of days with wind speed > 10 m s^−^^1^) in JJ23. **b**, The JJ23–JJ9015 difference in storminess index. Contours show 95% significance level. Fields are derived from the ERA5 reanalysis. Boxes in **b** are reproduced from Fig. 1 for reference and show the four main ice-decline regions.

The increased ocean heat loss may also be expected to affect the atmosphere, for example, the frequency of storms over previously ice-covered regions. A wide range of near and far-field atmospheric impacts (reaching to the Northern Hemisphere), on timescales from months to decades, has been shown in an earlier study through experiments using a forced low-resolution ocean–atmosphere coupled model^6^. These include changes to the eddy-driven westerly jet, occurring simultaneously with the ice reduction, which are greatest in austral winter. Here we focus on storms and ask whether there is observational evidence for an increase in storm occurrence in the regions of JJ23 enhanced ocean heat loss. Storm activity can be assessed using a wide range of metrics^25^. We use the threshold exceedance approach by taking the number of days with winds exceeding 10 m s^−1^ as a measure of storm frequency (Fig. 5; similar results are obtained using the MERRA-2 reanalysis instead of ERA5 and when using the 90th percentile of wind speed as an alternative storm metric; Extended Data Fig. 5). In the sea-ice-decline regions, the June–July storm frequency has increased by up to 7 days per month in 2023 relative to 1990–2015 (Fig. 5). The strongest increase is found in the centre of the northern Weddell Sea box, with further increases in the other three regions. The average of the storminess index over the four boxes is 11.6 days for JJ23 compared with 9.1 ± 1.0 days for JJ9015, that is, storminess has increased by 2.5 days in JJ23 relative to the 1990–2015 climatology.

Analysis of the relationship between the heat loss and storminess using JJ23 daily time series in two regions in which the storm signal is particularly strong (a sub-region of the northern Weddell Sea and the northern Ross Sea; Extended Data Figs. 6–8) shows that the integrated heat loss diverges from the climatological value at the start of June, whereas the number of storms stays close to climatology and begins to diverge at a lagged timescale of 3–5 days. This is consistent with the excess heat loss to the atmosphere leading to subsequent enhanced storm generation. Further analysis using storm tracking, together with atmosphere or coupled ocean–ice–atmosphere model experiments with ice decline mirroring that observed in 2023, is needed to fully determine the chain of cause and effect in the atmospheric response, including evaluation of the extent to which storminess is increased by the extra heat loss from reduced sea-ice coverage. Nevertheless, our results clearly indicate that the frequency of storms increased in JJ23. It is possible that our analysis includes other high-wind events as well as storms but, in both cases, the increased winds can have further consequences for the ocean, through storm-driven near-surface mixing, enhancement of heat loss and break-up of ice^26^.

Winter 2023 represents a markedly different state of ocean–atmosphere interaction for the previously ice-covered Southern Ocean than at any other time in the modern period. The anomalous exposure of the ocean surface resulting from the sea-ice decline was accompanied by an unprecedented doubling of the ocean to atmosphere heat supply. This abrupt strengthening is a marked change from the heat loss in both the stable 1990–2015 pre-ice-decline conditions and the ice-decline-onset phase, 2016–2022. It is too early to state whether 2023 marks the onset of a long-term regime shift but it is indicative of the extreme heat-loss winter conditions to be expected in future years of low ice regrowth.

On the basis of our results, such years will see an enhanced surface flux contribution to water-mass formation at high densities not previously produced in historically ice-covered regions. These regions are located away from the shelf edge areas in which the deepest water masses are formed but have the potential to influence shelf production, as advection and mixing will eventually bring some of the new offshore-water-mass classes onto the shelf, particularly in the Weddell and Bellingshausen seas. The increased winter 2023 ocean heat loss also has the potential to influence the atmospheric circulation and thus the wider climate system. We have shown that the record-breaking Antarctic sea-ice decline in 2023 has had a substantial impact on ocean–atmosphere interaction in the previously ice-covered regions of the Southern Ocean. Repeated low ice-cover conditions in subsequent winters will strengthen these impacts and are also likely to lead to profound changes further afield, including the tropics and the Northern Hemisphere.

## Methods

### Data sources

The surface flux, meteorological variables and sea-ice concentration presented in our study are from the European Centre for Medium-Range Weather Forecasts Reanalysis 5 (ERA5 (ref. ^27^)) reanalysis. The ERA5 sea-ice data are satellite-observation-derived fields from the EUMETSAT Ocean and Sea Ice Satellite Application Facility (OSI SAF) product^27^. Ocean surface salinity required for the water-mass-transformation calculation has been obtained from the UK Met Office EN.4.2.2 dataset^28^.

### Determination of box means and anomalies

Area-weighted means of various fields (sea-ice concentration, net heat flux and its components) have been determined for each of the four regions shown in Fig. 1. The weighting is carried out according to the area of individual ERA5 grid cells. Box boundaries are as follows: nWS, northern Weddell Sea (66° S to 59° S, 50° W to 20° E); BS, Bellingshausen Sea (75° S to 65° S, 85° W to 65° W); nwRS, northwestern Ross Sea (68° S to 62.5° S, 152° E to 175° E); EL, north of Enderby Land (66° S to 62.5° S, 40° E to 62° E). The combined four-box means are an average of the four individual box means weighted according to the area of each box. For ice concentration terminology, note, for example, that by use of the term 80% reduction in the main text, we mean a change in the fractional ice concentration of 0.8. All anomalies are determined with respect to the 30-year climatological reference period 1991–2020. Sea-ice-area values reported in the main text have been determined by multiplying the ERA5 sea-ice concentration (which varies in the range 0–100%) in a given grid cell by the grid-cell area and summing over all grid cells within the four boxed regions. For heat flux, the sign convention is for positive/negative heat flux to indicate ocean heat gain/loss to the atmosphere.

### Water-mass transformation and formation

Following standard water-mass-transformation theory^29^, the ERA5 evaporation (*E*), precipitation (*P*) and net heat flux into the ocean (*Q*) are used to estimate the surface-forced transformation (SFT) across an isopycnal, *σ*,

$${\rm{SFT}}({\sigma }^{* })=\frac{1}{\Delta \sigma }\iint \left[-\frac{\alpha }{{C}_{{\rm{P}}}}Q+\beta \frac{S}{1-S}(E-P)\right]\Pi (\sigma ){\rm{d}}x{\rm{d}}y$$

in which

$$\Pi (\sigma )=\left\{\begin{array}{cc}1 & {\rm{for}}\,|\sigma -{\sigma }^{* }|\,\le \,\frac{\Delta \sigma }{2}\\ 0 & {\rm{elsewhere}}\end{array}\right.$$

*α* is the thermal expansion coefficient, *β* is the haline contraction coefficient, *C*_P_ is the specific heat capacity at 5 m and *S* is the 5 m salinity. For each month and each isopycnal, *σ*, the local buoyancy flux (term in square brackets) is integrated over the surface area of the associated density bin, Δ*σ*. Thus, the SFT has a non-zero value for those months when the specified isopycnals outcrop, otherwise it is set to zero. In practice, the calculation is carried out using a discretized version of the above equation by summing over ERA5 grid cells within a particular density interval with a density bin size of Δ*σ* = 0.1 kg m^−^^3^. Formation is then determined as the difference in transformation values between the upper and lower limits of the density interval.

### Robust pattern of heat loss across reanalyses

The spatial patterns of JJ23 net heat-flux anomaly within the sea-ice zone determined from our primary reanalysis (ERA5) and the other leading, regularly updated reanalysis (MERRA-2 (ref. ^30^)) are shown in Extended Data Fig. 1a,b. The figure highlights the close similarity in the pattern of net heat-flux anomaly in both reanalyses, with strong heat losses in the boxed regions in each case and slightly higher values with MERRA-2. Thus, the pattern is robust to the choice of reanalysis, indicating that the intensity of the increased heat loss in JJ23 dominates any variations that may arise from differences in reanalysis physics and data assimilation. This is important to establish, as reanalyses can vary in their representation of air–sea interaction in the Southern Ocean, particularly the climatological mean heat exchange.

An evaluation of ERA5 against three drifting-buoy measurements in the Weddell Sea ice pack^31^ indicates that the reanalysis air temperature is close to the observations at 0 °C but develops a warm bias as temperatures decrease towards −40 °C. Such a bias would lead to the ERA5 heat-loss values underestimating the true heat loss and may account in part for the stronger losses seen with MERRA-2, but the close pattern agreement between the two reanalyses indicates that it does not strongly influence our conclusions. Note that MERRA-2 was not included in the buoy comparison study, so it is not possible to say whether it is more accurate than ERA5, as it may have a cold bias relative to the buoys and hence overestimate the true heat loss.

As well as ERA5 and MERRA-2, we have also examined the JJ23 net heat-flux anomaly with the NCEP/NCAR reanalysis^32^, which is another regularly updated reanalysis (Extended Data Fig. 1c). NCEP/NCAR is at a substantially coarser resolution than ERA5 and MERRA-2, as it was developed in the 1990s. Nevertheless, it still shows the main characteristics of the pattern found with the other two reanalyses, that is, increased heat loss in the main ice-decline regions, providing further support that this pattern is robust to the choice of reanalysis. Given the low resolution of the NCEP/NCAR fields, we subsequently focus on comparison of MERRA-2 results with ERA5.

### Variation of heat loss with sea ice

The variation of June–July air–sea heat flux with sea-ice concentration is shown in Extended Data Fig. 2. A clear relationship showing increasing heat loss with ice decline is evident and JJ23 is seen to fall a long way outside the group of all other points, emphasizing the exceptionally strong heat loss and low ice fraction in winter 2023. Typical values for the earlier years are sea-ice fraction in the range 0.6–0.8 and heat loss from −75 to −50 W m^−2^, whereas JJ23 has an ice fraction of only 0.4 and increased heat loss at −90 W m^−2^. Although marked ice reductions are known to have occurred within the 2016–2022 period, they were mostly in the austral summer sea-ice extent (for example, refs. ^3,9,17^) rather than austral winter. The JJ1622 values are seen to be shifted towards the lower end of the distribution for JJ9015 but are not clearly separated from them, unlike JJ23.

### Climatological and JJ23 heat flux

**Extended Data Fig. 3:**
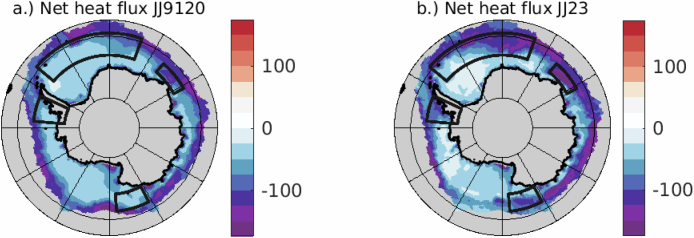
Comparison of mean June–July net heat flux in 2023 with climatology reveals stronger heat loss in 2023. ERA5 mean net heat flux for June–July climatology (1991–2020) (**a**) and JJ23 (**b**), units W m^−^^2^. Sign convention is for positive/negative heat flux to indicate ocean heat gain/loss to the atmosphere. Black boxes show the regions defined in the main text.

To aid interpretation of the JJ23 net heat-flux-anomaly map shown in Fig. 1b, we present mean heat-flux maps for both the climatological case (1991–2020) and for 2023 (Extended Data Fig. 3). The two maps show that, in both cases, the June–July net heat flux ranges from near zero to strongly negative (heat loss approaching −200 W m^−2^) over the sea-ice region, with strongest losses in the outer half towards the ice edge. Comparison of the maps reveals stronger heat loss in the boxed regions in 2023 compared with climatology. Consideration of Fig. 1b reveals that the negative heat-loss anomaly in the Ross Sea box is different in character to the other three boxes, as it is accompanied by a narrow band of positive heat-flux anomaly along the outer ice edge to the northeast of the box. This positive anomaly can be seen through comparison of Fig. 1b and Extended Data Fig. 3 to reflect a reduction in the normally strong heat loss in this band. For the wider Ross Sea region, the positive and negative anomalies in Fig. 1b have the potential to cancel out, but this does not affect the conclusions reached for the northern Ross Sea box as defined in our analysis.

### Water-mass formation across reanalyses

**Extended Data Fig. 4:**
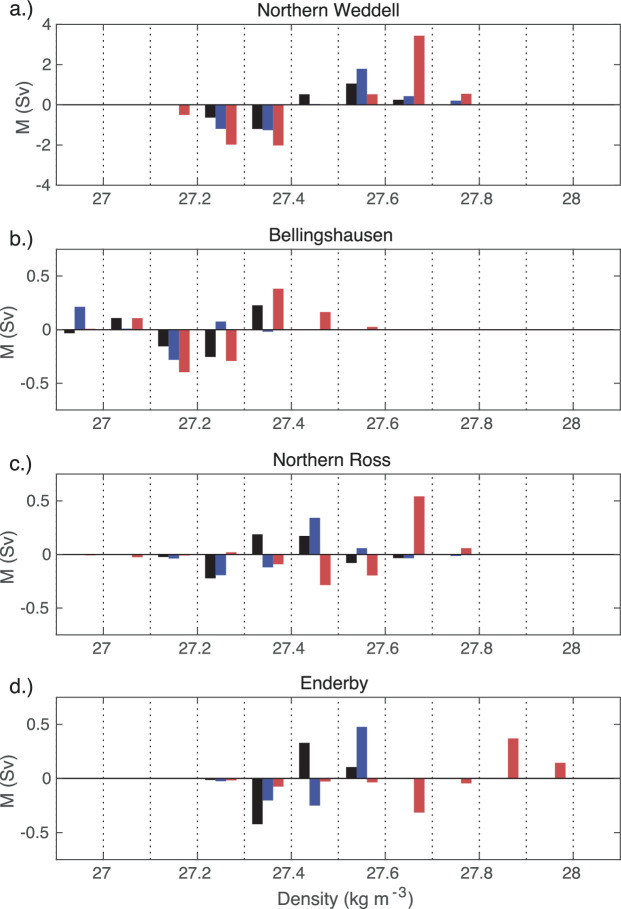
Comparison of surface flux contribution to water-mass formation (*M*) in the pre-ice-decline and ice-decline periods obtained with MERRA-2 shows close agreement with main-text ERA5 results. Counterpart figure obtained using fields from the MERRA-2 reanalysis to Fig. 4 obtained with ERA5. Values are shown in 0.1 kg m^−^^3^ class intervals for JJ9015 (black), JJ1622 (blue) and JJ23 (red). Units are Sv, in which 1 Sv = 10^6^ m^3^ s^−1^. **a**, Northern Weddell Sea. **b**, Bellingshausen Sea. **c**, Northwestern Ross Sea. **d**, Enderby. Note the difference in *y*-axis scales between **a** and **b**–**d**.

The main-text analysis of the surface flux contribution to water-mass formation in the pre-ice-decline and ice-decline periods determined from our primary reanalysis (ERA5) has been repeated using MERRA-2 (Extended Data Fig. 4). Comparison of Extended Data Fig. 4 with Fig. 4 shows the close similarity between the two sets of results, that is, the stronger high-density contribution to June–July water-mass formation in 2023 than both 1990–2015 and 2016–2022 is seen with both reanalyses. The only difference of note is in the Weddell Sea, in which the MERRA-2 JJ23 water-mass-formation anomalies are stronger than ERA5 (note the difference in *y*-axis range between Extended Data Fig. 4 and Fig. 4a), consistent with the increase in heat loss with MERRA-2 noted in the discussion of Extended Data Fig. 1. Otherwise, Extended Data Fig. 4 is virtually indistinguishable from Fig. 4. This demonstrates the robustness to variations in reanalysis physics and data assimilation of our result that the surface flux contribution to JJ23 water-mass formation has undergone a notable shift to higher density classes. Further insights into changes in the ice-decline regions, including the processes underlying water-mass formation and the balance of changes in ice cover and heat flux in setting the temperature and salinity fields, are anticipated from analysis of ocean-state estimates for 2023 when they become available.

### Increase in storminess across reanalyses

**Extended Data Fig. 5:**
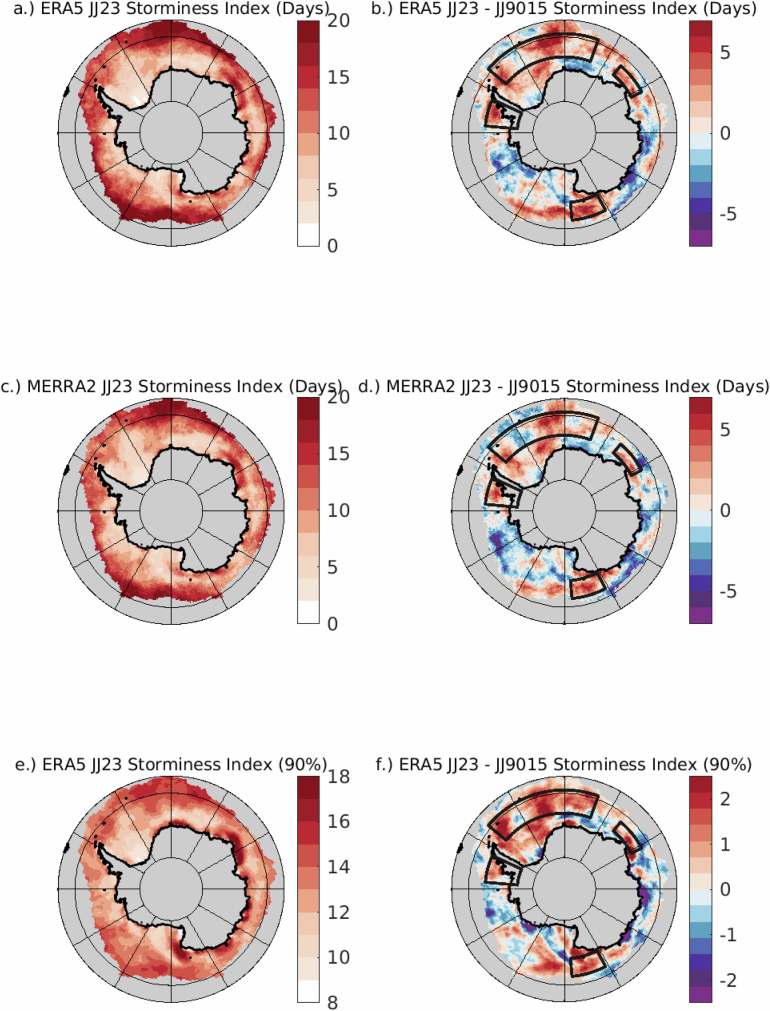
Comparison of winter 2023 storminess relative to 1990–2015 with ERA5 and MERRA-2 shows close agreement. **a**. ERA5 storminess index (number of days with wind speed > 10 m s^−1^) in JJ23. **b**, The ERA5 JJ23–JJ9015 difference in storminess index. **c**,**d**, As per **a**,**b** but for MERRA-2. **e**,**f**, As per **a**,**b** but using an ERA5 storminess index defined by the 90th percentile of wind speed (units m s^−^^1^). Boxes show the four regions used for the main-text analysis.

The increase in storminess in June–July of 2023 relative to 1990–2015 obtained from ERA5 and reported in the main text (Fig. 5 and associated discussion) has been recalculated using the MERRA-2 reanalysis (Extended Data Fig. 5c,d). Comparison of the ERA5 and MERRA-2 fields for both the JJ23 storminess and the JJ23–JJ9015 storminess difference shows very similar results. In both cases, the June–July storm frequency has increased by up to 7 days per month in 2023 in the sea-ice-decline regions. Thus, our conclusions about the storminess increase are not sensitive to differences in reanalysis physics and data assimilation. Note also that the increased storminess is not confined to only the high sea-ice-decline areas, in particular, there are strong increases to the east of the Ross Sea box. This may indicate broader-scale impacts of the sea-ice decline on the atmospheric circulation that warrant further analysis using atmospheric-model experiments.

The robustness of our choice of primary wind speed metric (number of days above a fixed 10 m s^−1^ threshold) has been assessed using an alternative index, the 90th percentile of wind speed determined on a grid cell by grid cell basis. The results shown in Extended Data Fig. 5e,f are very similar to those obtained with the fixed threshold. Thus, both the 10 m s^−^^1^ threshold and the 90th percentile wind-speed-based metrics indicate an increase in storminess. These metrics-based results motivate further analyses in subsequent research, in particular, the use of storm-tracking algorithms to investigate impacts on trajectories as well as frequency and coupled-model analysis to study the atmospheric response in detail.

### Lag between heat loss and storms

**Extended Data Fig. 6:**
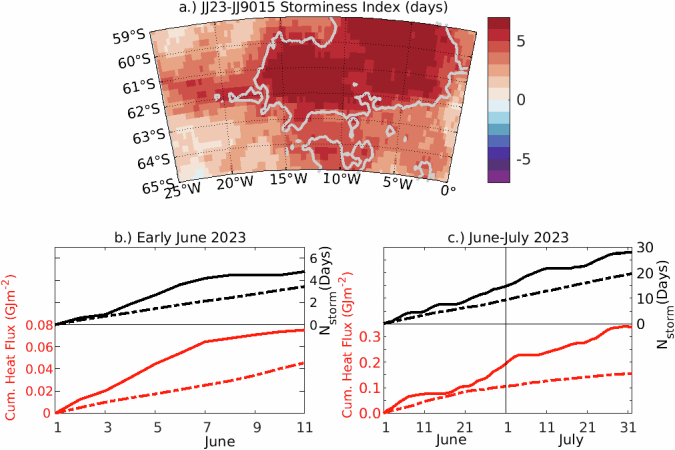
Map and comparison of cumulative ERA5 turbulent heat flux and storm occurrence time series in JJ23 for the northern Weddell Sea sub-region. **a**, Difference in storminess index between JJ23 and JJ9015 for the northern Weddell Sea sub-region. Grey contour shows 95% significance. **b**, Sub-region cumulative time series for 1–10 June 2023 of turbulent heat flux (solid red line, 2023 values; dashed red line, 1991–2015 climatology) and storm number (solid black line, 2023 values; dashed black line, 1991–2015 climatology). **c**, As **b** but for JJ23. Time series are area-weighted means formed by averaging over all individual grid cells within the box. Heat-flux units are GJ m^−^^2^, in which 1 GJ m^−^^2^ = 10^9^ J m^−^^2^. Number of storm days has been determined using the threshold exceedance approach with a threshold of 10 m s^−^^1^.

We have carried out further analysis of the relationship between heat loss and storminess using daily time series of the net turbulent heat flux within the JJ23 period (Extended Data Fig. 6). For the analysis, a sub-region (65° S to 59° S, 25° W to 0° E) of the northern Weddell Sea has been selected (Extended Data Fig. 6a), which has a strong increase in storm number. The 95% significance level contour encloses much of the sub-region, that is, there is robust coverage of the sub-region by increased storm number. Cumulative turbulent heat flux and storm occurrence time series for 1–10 June 2023 are shown in Extended Data Fig. 6b, with an extended version for the whole of JJ23 in Extended Data Fig. 6c. A delayed increase in storm number with respect to the increase in heat flux on a timescale of approximately 3 days is evident in Extended Data Fig. 6b. This result is consistent with a scenario in which the increased heat supply to the atmosphere results in an increase in storm frequency. By the end of July, the number of storm days is close to 30, nearly 50% higher than the value of 20 seen for climatology (Extended Data Fig. 6c).

**Extended Data Fig. 7:**
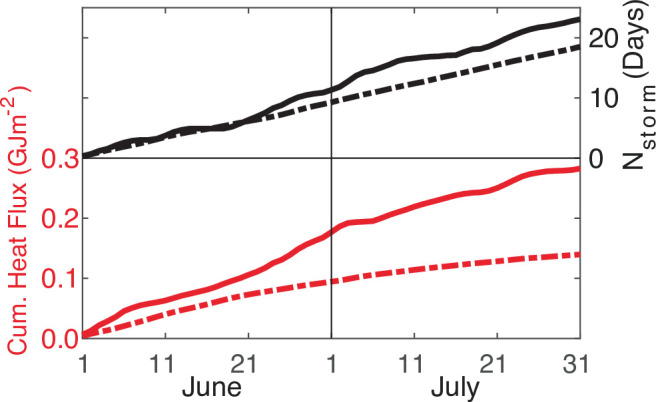
Comparison of cumulative ERA5 turbulent heat flux and storm occurrence time series in JJ23. The time series are area-weighted means for the northern Weddell Sea box formed by averaging over all individual grid cells within the box. Line colour and style as per Extended Data Fig. 6. Heat-flux units are GJ m^−^^2^, in which 1 GJ m^−^^2^ = 10^9^ J m^−^^2^. Number of storm days has been determined using the threshold exceedance approach with a threshold of 10 m s^−^^1^.

The analysis has been repeated for the whole northern Weddell Sea box (largest box in Fig. 1b), which contains regions of both strong and weak heat-loss increase. In this case, a longer delay in storm increase is observed for the whole box of about 20 days (Extended Data Fig. 7). This is to be expected, as the rapid 3-day signal seen in the sub-region will be extended by the inclusion of the remainder of the region, which includes areas of weaker increase in heat loss that are likely to take longer to experience a storm response or may not respond at all.

**Extended Data Fig. 8:**
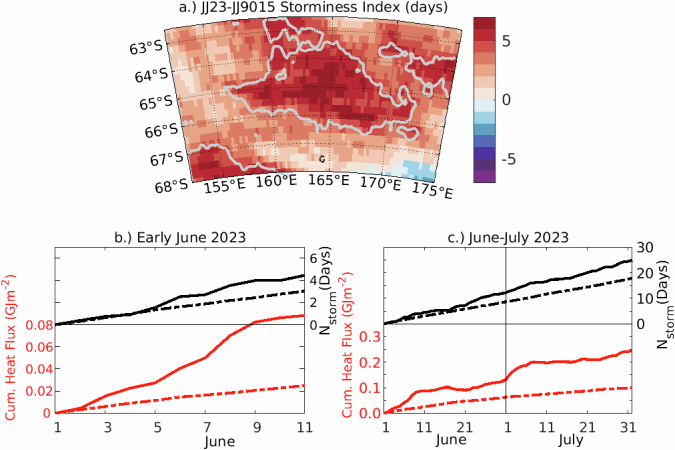
Map and comparison of cumulative ERA5 turbulent heat flux and storm occurrence time series in JJ23 for the northern Ross Sea region. **a**, Difference in storminess index between JJ23 and JJ9015 for the northern Ross Sea region. Grey contour shows 95% significance. **b**, Sub-region cumulative time series for 1–10 June 2023 of turbulent heat flux (solid red line, 2023 values; dashed red line, 1991–2015 climatology) and storm number (solid black line, 2023 values; dashed black line, 1991–2015 climatology). **c**, As **b** but for JJ23. Time series are area-weighted means formed by averaging over all individual grid cells within the box. Heat-flux units are GJ m^−^^2^, in which 1 GJ m^−^^2^ = 10^9^ J m^−^^2^. Number of storm days has been determined using the threshold exceedance approach with a threshold of 10 m s^−^^1^.

We have repeated the northern Weddell Sea analysis for the northern Ross Sea box, shown in Fig. 5 and reproduced at larger scale in Extended Data Fig. 8. This is another region of strong increase in the storminess index in JJ23 (Extended Data Fig. 8a). When averaged over the box, the time series again show a rapid response of storminess that, in the northern Ross Sea, lags the heat-flux increase by 4–5 days (Extended Data Fig. 8b,c).

Further work using model-based experiments to explain the underlying causal processes responsible for the lagged response of storminess to increasing ocean heat loss is desirable. Such a study would require careful consideration of model experiment design, including whether a coupled ocean–atmosphere model is necessary rather than a forced atmospheric model and the need to carry out a sufficiently large ensemble of model control and perturbed simulations to be able to draw robust conclusions. We would hope to see such a study stimulated by the results of our paper

In summary, we find evidence for a short-timescale (3–5-day lag) increase in storm number following the increase in heat loss in both the northern Weddell Sea sub-region and the northern Ross Sea (Extended Data Figs. 6b and 8b). A rapid response on this timescale is consistent with a causal relationship in which the heat loss drives the storm increase. Note that this result for within-season variation stands in contrast to drivers of multidecadal trends in which variations in the wind field and storminess have been found to control the sea-ice extent^33,34^ and so, potentially, the heat loss.

### Variation of *M* and storms with heat loss

**Extended Data Fig. 9:**
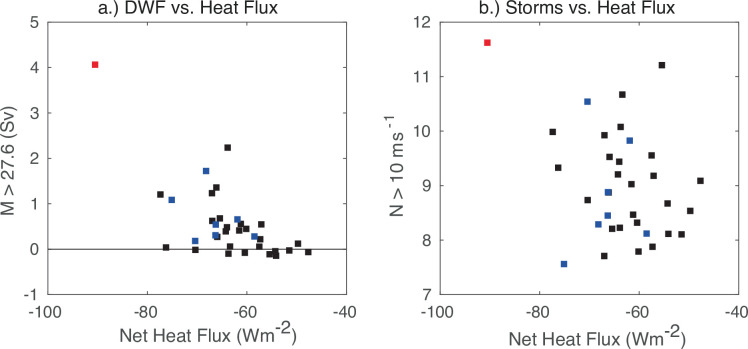
Variation of dense water formation (DWF) and number of storms with net air–sea heat flux. **a**, Values shown are the water-mass formation (*M*) at densities >27.6 kg m^−^^3^ summed over the four boxed ice-decline regions defined in the main text. Colours denote June–July means for 1990–2015 (black), 2016–2022 (blue) and 2023 (red). **b**, Values shown are the number of storms (*N*, obtained using the 10 m s^−^^1^ exceedance threshold) averaged over the four boxed ice-decline regions. Colours as per **a**. Data source: ERA5 reanalysis.

The variation of June–July water-mass formation (*M*) at densities >27.6 kg m^−^^3^ summed over the four ice-decline regions with air–sea heat flux is shown in Extended Data Fig. 9a. The amount of water formed in this density range in JJ23 (4.1 Sv) is nearly twice as large as that found in the strongest of the earlier years (2.2 Sv), indicating the exceptional water-mass-formation conditions that took place in 2023. In many of the early years, little or no water was formed at densities >27.6 kg m^−^^3^ and, in some years, small negative values indicate a net lightening of water in this density range, rather than the strong densification seen in 2023.

The variation of June–July number of storms averaged over the four ice-decline regions with air–sea heat flux is shown in Extended Data Fig. 9b. The number of storms in JJ23 exceeds that in June–July of all other years, although the separation is less clear cut than for dense water formation. The JJ23 value is 11.6 days compared with 9.1 ± 1.0 days for 1990–2015, that is, the 2023 value is significantly different at the 95% level.

### Annual heat-loss anomalies

**Extended Data Fig. 10:**
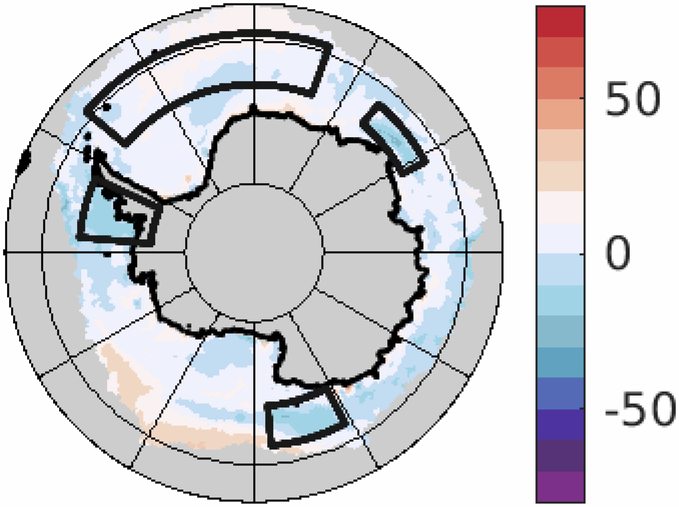
ERA5 annual mean turbulent heat-flux anomaly for 2023. Anomalies are determined relative to 1991–2020, units W m^−2^. Boxes show the four regions used for the main-text analysis. Colour scale is chosen to be the same as Fig. 1.

The main focus of our study is the extreme winter heat loss in JJ23, but we have also carried out some more analysis for the year as a whole. A map of the annual 2023 turbulent heat-flux anomalies is shown in Extended Data Fig. 10. The flux anomalies are typically less than 10 W m^−2^, although stronger negative values in the range 20–30 W m^−2^ are evident in the Bellingshausen Sea, northwestern Ross Sea and Enderby Land regions. The northern Weddell Sea tends to exhibit rather weak anomalies over the whole year, in contrast to the strong winter anomalies described in the main text, and we plan further investigation of the reasons for this difference in subsequent work. We have also calculated the annual mean values for the different curves in Fig. 3 and find that the 2023 annual mean value is not statistically different from climatology.

## Online content

Any methods, additional references, Nature Portfolio reporting summaries, source data, extended data, supplementary information, acknowledgements, peer review information; details of author contributions and competing interests; and statements of data and code availability are available at 10.1038/s41586-024-08368-y.

## Data Availability

Data used in this study are available as follows: ERA5, https://cds.climate.copernicus.eu/datasets/reanalysis-era5-single-levels-monthly-means?tab=download; EN.4.2.2, https://www.metoffice.gov.uk/hadobs/en4/download.html; MERRA-2 (ref. ^30^), https://disc.gsfc.nasa.gov/datasets?project=MERRA-2.

## Code availability

Code used in this study are available from the Zenodo repository (10.5281/zenodo.13971927)^35^.
